# Low humoral immune response to the BNT162b2 vaccine against COVID-19 in nursing home residents undergoing hemodialysis: a case–control observational study

**DOI:** 10.1186/s41100-022-00397-5

**Published:** 2022-03-16

**Authors:** Mineaki Kitamura, Takahiro Takazono, Kazuko Yamamoto, Takashi Harada, Satoshi Funakoshi, Hiroshi Mukae, Tomoya Nishino

**Affiliations:** 1 Nagasaki Renal Center, Nagasaki, Japan; 2 Department of Nephrology, Nagasaki University Graduate School of Biomedical Sciences, 1-7-1 Sakamoto, Nagasaki, 852-8501 Japan; 3 Department of Infectious Diseases, Nagasaki University Graduate School of Biomedical Sciences, Nagasaki, Japan; 4 Department of Respiratory Medicine, Nagasaki University Hospital, Nagasaki, Japan; 5 Department of Respiratory Medicine, Nagasaki University Graduate School of Biomedical Sciences, Nagasaki, Japan

**Keywords:** COVID-19, Hemodialysis, Humoral response, Immunogenicity, SARS-CoV-2, Vaccination

## Abstract

**Background:**

Patients on hemodialysis (HD) face a high mortality risk from coronavirus disease 2019 (COVID-19), caused by severe acute respiratory syndrome coronavirus 2 (SARS-CoV-2), and they are therefore prioritized for vaccination. However, the efficacy of vaccination in this vulnerable population has not been confirmed. Although age is negatively correlated with serum immunoglobulin (Ig) levels, humoral responses to vaccination in elderly patients undergoing HD have not been investigated. To address this issue, we evaluated the anti-SARS-CoV-2 spike protein antibodies in nursing home residents on HD after BNT162b2 vaccine administration.

**Methods:**

Patients on HD from a nursing home and care workers (controls) receiving two doses of the BNT162b2 vaccine between April and May 2021 were enrolled in this study. Those with a prior history of COVID-19 were excluded. Anti-spike protein antibodies were measured with the Elecsys (Roche) immunoassay system.

**Results:**

The study included 26 nursing home residents (41% male; median age, 86 years) and 184 care workers (28% male; median age, 45 years). The median HD vintage was 51 months. After two doses of BNT162b2, 73% of the nursing home residents and 99.5% of the control group developed sufficient anti-spike protein antibodies (> 29 U/mL) to neutralize SARS-CoV-2. Three weeks after the second dose, median IgG titers of the residents and care workers were 83 [interquartile range (IQR) 17–511] and 1365 (IQR 847–2245) U/mL, respectively (*p* < 0.001).

**Conclusions:**

The humoral response to BNT162b2 among elderly HD patients was relatively low; therefore, the optimal vaccination strategy for this population should be studied further to avoid COVID-19 outbreaks in healthcare facilities.

**Supplementary Information:**

The online version contains supplementary material available at 10.1186/s41100-022-00397-5.

## Background

The prevention of coronavirus disease 2019 (COVID-19), caused by severe acute respiratory syndrome coronavirus 2 (SARS-CoV-2) is a crucial issue for patients on hemodialysis (HD) [1], as patients receiving in-center HD are at an increased risk of contracting the disease. Similarly, elderly people who live in nursing homes are at a high risk of contracting COVID-19 [2], and patients on HD who live in nursing homes are particularly susceptible to the disease and associated complications (odds ratio, 6.92) [3]. Thus, residents or outpatients have a high mortality risk once a COVID-19 outbreak occurs in nursing homes and HD centers [4]. Vaccination plays a key role in preventing COVID-19; however, there is a concern that the efficacy of vaccines among patients undergoing HD may be lower than that for the general population [5].

Effective and safe vaccines are needed to curb the COVID-19 pandemic; fortunately, effective anti-COVID-19 mRNA vaccines are available. However, despite the high efficacy of BNT162b2 (Pfizer-BioNTech), emerging evidence indicates that certain populations do not benefit from adequate immunity against COVID-19, including nursing home residents; patients with hereditary immunodeficiency, malignancy, or diabetes mellitus; patients with human immunodeficiency virus; and patients on immunosuppressive therapies [6, 7]. Moreover, patients on HD show generally low immune responses and poor humoral responses to vaccines [8, 9]. For example, the first dose of BNT162b2 can induce adequate immunoglobulin G (IgG) generation in more than half of the general population, whereas only 35% of patients undergoing HD develop adequate immunity [10]. Further, the IgG titer following the second dose has been reported to be lower in patients on HD than in healthy controls [11, 12].

Recently, there has been a continuous increase in the number of elderly people on dialysis therapies, some of whom also live in nursing homes [13]. Given the susceptibility of this population to contracting COVID-19, it is crucial for elderly patients on HD to produce adequate levels of IgG against COVID-19 after vaccination to confer sufficient protection. Unfortunately, despite the known risks, there is little evidence regarding BNT162b2 vaccine effectiveness for elderly patients on HD. Therefore, we aimed to elucidate BNT162b2 efficacy in elderly patients on HD, focusing on nursing home residents.

## Methods

### Study participants

Between April and May 2021, residents of a nursing home specific to patients undergoing HD (Kokura-an, Nagasaki City, Japan) and healthcare workers from Kokura-an and Nagasaki Renal Center (as a control group) who received two doses of BNT162b2 (30 μg intramuscular injection) were included in this study. Due to the availability of data, healthcare workers were determined as the control, in line with previous studies [11, 12, 14]. All nursing home residents were undergoing HD at Nagasaki Renal Center. The second dose of BNT162b2 was administered 21 days after the first dose.

Exclusion criteria included patients and healthcare workers with a prior history of COVID-19, healthcare workers who did not have their anti-spike protein antibodies measured and had undergone kidney transplantation, and individuals who did not consent to be included in the study. All other participants provided verbal informed consent for inclusion. In addition, a patient who died before the IgG level could be measured 3 weeks after the second dose was also excluded. This study was approved by the ethics committee of Nagasaki Renal Center (Nagasaki, Japan) (approval number: 21005) and was performed according to the 1964 Declaration of Helsinki and its later amendments.

### Anti-spike protein measurement

Given the high risks for this population, the levels of anti-spike protein antibodies against SARS-CoV-2 were measured in patients and healthcare workers after they received anti-COVID vaccination at the two facilities to prevent a COVID-19 outbreak. Anti-spike protein antibodies were measured using the Elecsys® Anti-SARS-CoV-2 immunoassay (Roche Diagnostics International Ltd., Switzerland) at three time points, 3 weeks after the first injection, and at 2 and 3 weeks after the second injection, respectively. The “lower detection limit” and “cut-off level for a seronegative result” were 0.4 U/mL and 0.8 U/mL, respectively. An effective protective IgG level was determined to be ≥ 29 U/mL [14] based on the reported virus-neutralization ability [15]. Furthermore, we tested the presence of nucleocapsid (*N*) antibodies using the Elecsys® assay to exclude subjects with a COVID-19 history [cut-off index < 1.0 interpreted as non-reactive (negative)]. Patient background data were obtained from their medical records, and routine blood test results were obtained just before the first dose of BNT162b2 was administered as part of routine clinical practice. Blood test results and body mass index of healthcare workers were obtained during the routine health check.

### Statistical analysis

Categorical variables are expressed as numbers (%), whereas continuous values are expressed as medians with their respective interquartile ranges (IQRs). Wilcoxon rank-sum and chi-square tests were used to evaluate differences between the two study groups. Spearman analysis was used to calculate correlations between two continuous variables, and correlation coefficients are shown as *ρ* values. For the statistical analysis, if the anti-spike protein antibody value was below the detection limit (0.4 U/mL), it was given a value of 0.39 U/mL, according to a previous report [16]. Body mass index was calculated using the patients’ dry weight and height (kg/m^2^). A *p* value of < 0.05 was considered statistically significant. Statistical analyses were performed using JMP 15 software (SAS Institute Inc., Cary, NC, USA).

## Results

Twenty-six nursing home residents undergoing HD and 184 healthcare workers were included in this study. One nursing home resident was excluded following death due to chronic illness after the second dose of BNT162b2, and seven healthcare workers were excluded as their antibody levels were not measured or because of a history of kidney transplantation. Two healthcare workers were excluded owing to a prior history of COVID-19. These two excluded care workers tested positive for nucleocapsid antibodies in their blood, whereas all other study participants tested negative. The median age of the nursing home residents was 86 years (IQR 77–90); 41% were male. The most common cause of renal failure in the nursing home residents was benign nephrosclerosis. None of the residents had received oral steroids or immunosuppressive drugs. The detailed background characteristics of the participants are shown in Table 1.

**Table 1:** Characteristics of nursing home residents on maintenance hemodialysis and healthcare workers

	Nursing home residents (*n* = 26)	Control group (*n* = 184)	*p* value
Age (years)	86 (77–90)	45 (34–55)	< 0.001
Sex (male) (number, %)	10, 38%	52, 28%	0.32
Body mass index (kg/m^2^)	19.6 (18.0–21.7)	21.7 (19.4–24.6)	0.002
Dialysis vintage (months)	62 (28–119)	NA	
Diabetes mellitus (number, %)	4, 15%	9, 5%	0.03
Hypertension (%)	21, 81%	13, 7%	< 0.001
History of ischemic heart diseases (number, %)	9, 34%	0, 0%	< 0.001
History of stroke (number, %)	7, 27%	0, 0%	< 0.001
Dementia (number, %)	17, 65%	0, 0%	< 0.001
Mean KT/V	1.55 (1.40–1.83)	NA	
White blood cell count (/μL)	5185 (4693–6065)	6155 (5310–7180)	< 0.001
Hemoglobin (g/dL)	10.6 (9.9–11.7)	13.5 (12.7–14.6)	< 0.001
Blood urea nitrogen (mg/dL)	52 (48–67)	13 (11–15)	< 0.001
Creatinine (mg/dL)	6.7 (5.9–7.7)	0.6 (0.5–0.7)	< 0.001
Albumin (g/dL)	3.2 (3.0–3.3)	NA	

*NA* not available

**Fig. 1:**
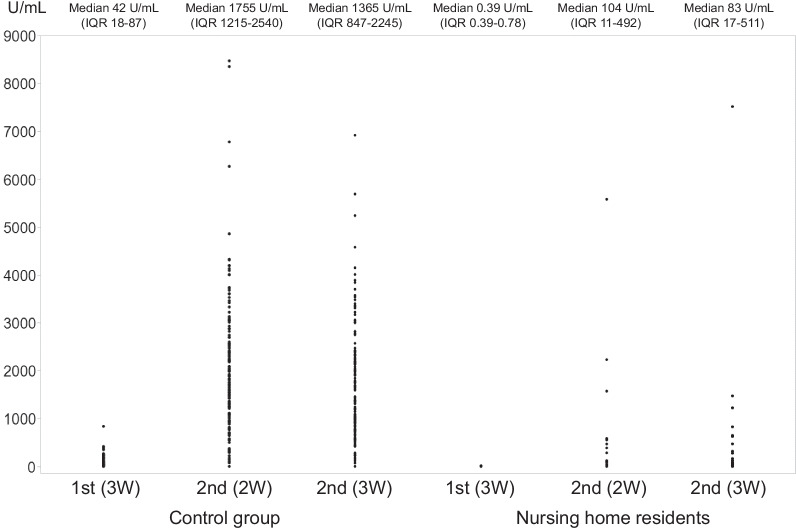
Levels of anti-spike protein immunoglobulin G (IgG) antibody against SARS-CoV-2 in healthy control and nursing home residents at 3 weeks after the first dose, 2 weeks after the second dose, and 3 weeks after the second dose. Compared to the healthy control, the nursing home residents showed lower IgG level at each point (*p* < 0.001)

**Fig. 2:**
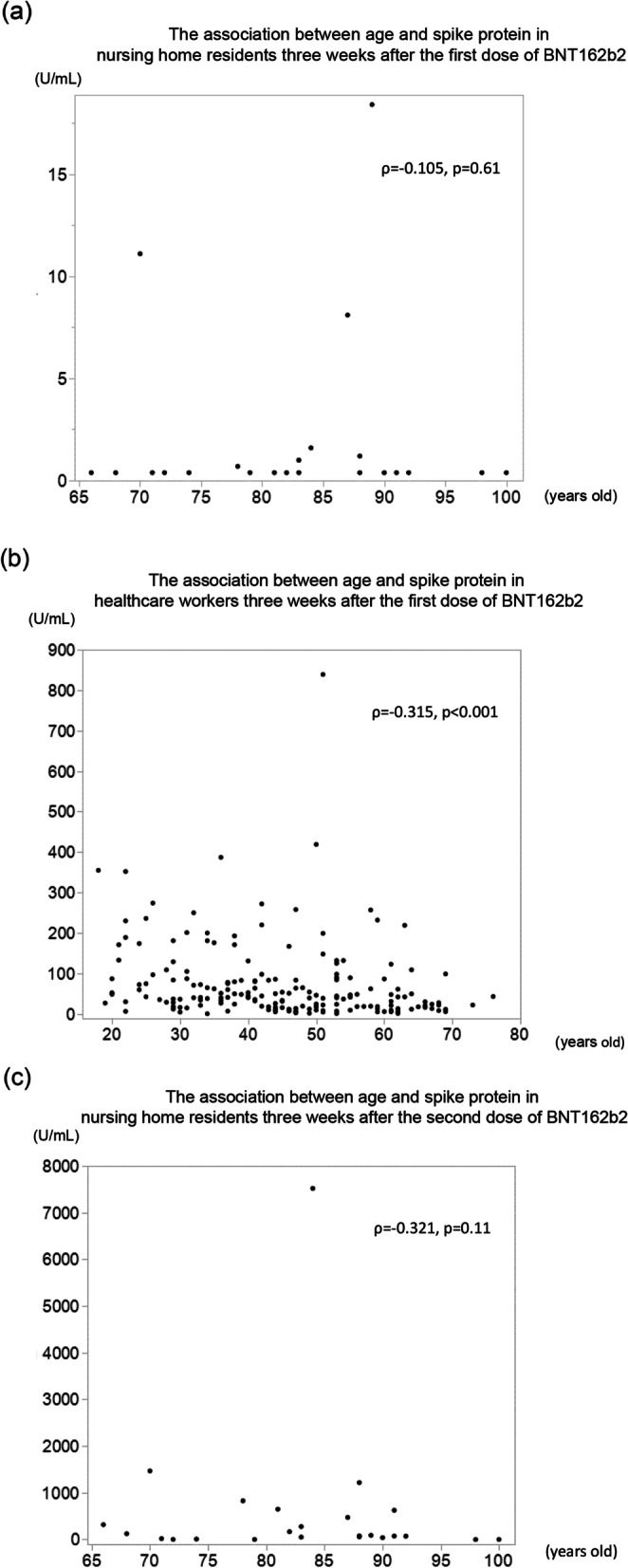

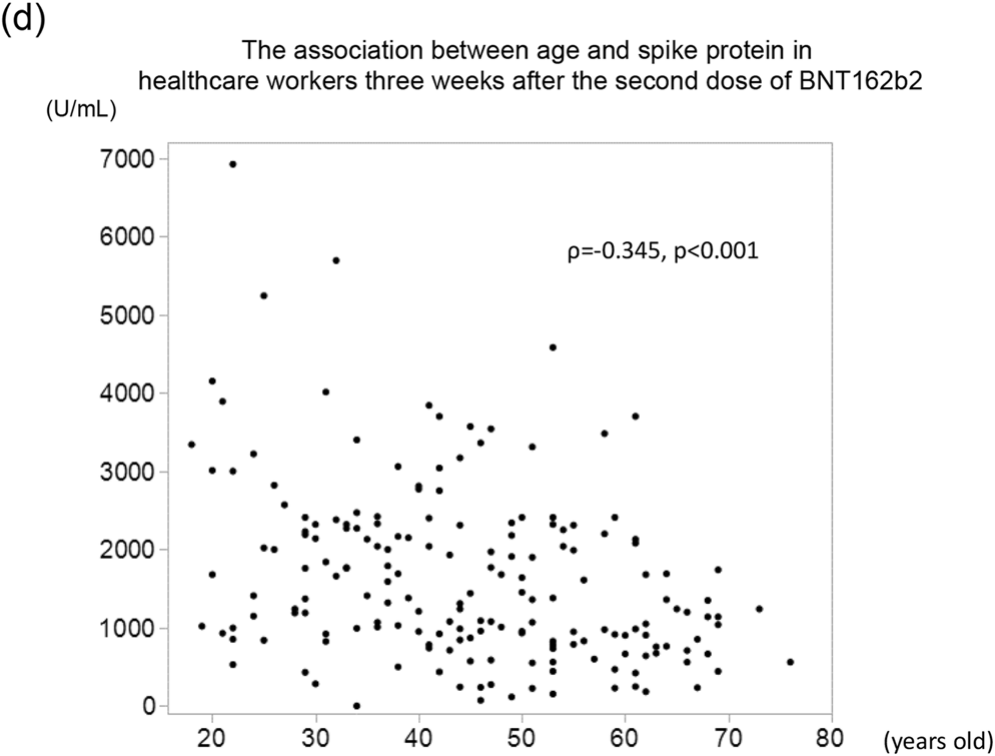
Correlation of age and anti-spike protein antibody levels in **a** nursing home residents 3 weeks after the first dose, **b** healthcare workers (control group) 3 weeks after the first dose, **c** nursing home residents 3 weeks after the second dose, and **d** healthcare workers (control group) 3 weeks after the second dose

Anti-spike protein antibodies in the subjects’ blood were analyzed 3 weeks after the first dose of BNT162b2. Only 6 (22%) nursing home residents on HD were confirmed to be seropositive, whereas 183 out of 184 (99.5%) of the healthcare workers in the control group were confirmed to be seropositive (*p* < 0.001). Notably, IgG antibodies could not be detected in 20 nursing home residents 3 weeks after the first dose (< 0.4 U/mL). By contrast, the median IgG level in the control group was 42 U/mL (IQR 18–87). There was no significant correlation between age and IgG titer in the nursing home residents (*ρ* =  − 0.105, *p* = 0.61), whereas this correlation was significant in the control group (*ρ* = − 0.315, *p* < 0.001) (Table 2, Figs. 1, 2).

**Table 2:** Humoral immune response to BNT162b2 vaccination according to serum IgG anti-spike protein antibody levels

	Nursing home residents (*n* = 26)	Control group (*n* = 184)	*p* value
Seronegative 3 weeks after the 1st dose (< 0.8 U/mL) (number, %)	20, 77%	1, 0.5%	< 0.001
Seronegative 2 weeks after the 2nd dose (< 0.8 U/mL) (number, %)	4, 15%	0, 0%	< 0.001
Seronegative 3 weeks after the 2nd dose (< 0.8 U/mL) (number, %)	2, 8%	0, 0%	0.004

At 2 and 3 weeks after the second dose, anti-spike protein antibody levels increased substantially in both groups (Fig. 1). Almost all the healthcare workers in the control group developed sufficient IgG levels at 2 and 3 weeks after the second dose (99% and 99.5%, respectively), whereas the percentages of the nursing home residents with sufficient IgG levels at the same points were 69% (*p* < 0.001) and 73% (*p* < 0.001), respectively. The association between age and IgG titer in the control group 2 weeks after the second dose was statistically significant (*ρ* = − 0.332, *p* < 0.001), whereas no such association was observed in the nursing home residents (*ρ* = − 0.289, *p* = 0.15). Consistently, there was no significant correlation between anti-spike protein antibodies and age in the nursing home residents (*ρ* = − 0.321, *p* = 0.11), whereas this correlation remained significant in the control group 3 weeks after the second dose (*ρ* = − 0.345, *p* < 0.001) (Fig. 2). The median antibody levels at 3 weeks after the second dose decreased compared with those measured 1 week before in both groups, but the week before in both the nursing home residents and the control group (54% (14/26) of the nursing home residents and 16% (30/185) of the healthcare workers showed an increased level of anti-spike protein antibodies (Fig. 1). There was no significant difference between patients with increasing (*n* = 14) and decreasing or unchanged IgG titers (*n* = 12) from 2 weeks after the second dose to 3 weeks after the second dose (data not shown).

There were no significant differences in age, sex, dialysis vintage, and other blood examination data between the nursing home residents who did not develop effective IgG levels (< 29 U/mL, *n* = 8) and those who developed sufficient antibodies (≥ 29 U/mL, *n* = 18) 2 weeks after the second vaccine dose (Additional file 1: Table S1). Four nursing home residents were seronegative (< 0.8 U/mL), and their ages (*p* = 0.04) and serum albumin levels (*p* = 0.045) were significantly higher than those who were seropositive (≥ 0.8 U/mL) at that time (Table 3). No severe adverse events such as anaphylaxis were observed in any study participant after injecting BNT162b2.

**Table 3:** Difference in background characteristics of seropositive and seronegative nursing home residents 2 weeks after the second dose

	Seropositive patients (*n* = 22)	Seronegative patients (*n* = 4)	*p* value
Age (years)	83 (73–88)	98 (84–100)	0.04
Sex (male) (number, %)	8, 36%	2, 50%	0.61
Body mass index (kg/m^2^)	20.4 (17.3–22.1)	18.6 (17.8–19.2)	0.27
Dialysis vintage (months)	51 (26–119)	96 (52–121)	0.37
Diabetes mellitus (number, %)	4, 18%	0, 0%	0.23
Mean KT/V	1.65 (1.40–1.92)	1.20 (1.30–1.65)	0.35
White blood cell count (/μL)	5110 (4590–5940)	6370 (5380–7280)	0.08
Hemoglobin (g/dL)	10.6 (9.9–11.8)	10.0 (9.7–10.5)	0.36
Blood urea nitrogen (mg/dL)	52 (49–69)	48 (33–62)	0.27
Creatinine (mg/dL)	6.7 (5.8–7.7)	7.2 (6.1–9.8)	0.55
Albumin (g/dL)	3.2 (3.1–3.4)	3.0 (2.7–3.1)	0.045

## Discussion

Our results showed that around 70% of the nursing home residents receiving HD developed sufficient anti-spike protein antibodies to be protected against COVID-19, even after receiving the second dose. Although the majority of the nursing home residents developed a significant humoral response to the second dose, the IgG titers of the anti-spike protein against SARS-CoV-2 were lower than those in the control group.

Elderly people with COVID-19 face a higher risk of death than young, healthy people [17]. The mortality rate of patients on dialysis is particularly high if they contract COVID-19 [18], as they are immunodeficient; thus, the infection will easily aggravate their general condition. Although vaccination for these vulnerable populations has been prioritized worldwide [8], there is a concern that vaccination efficacy might be limited in such groups due to weakened immune responses.

This study focused on elderly people undergoing HD as an extremely immunodeficient population at high risk of severe COVID-19 with a generally poor vaccine immune response. Indeed, we found that the IgG titer in these nursing home residents after the first vaccine dose was extremely low, and 20 of these 26 patients (77%) were seronegative. By contrast, only one healthcare worker (0.5%) (control group) was seronegative after the first dose. This finding is in line with a previous report showing that the seronegative rate after the first BNT162b2 dose in patients on HD (mean age, 68 years) was 65% [10]. Thus, the higher seronegative rate in our study is attributed to the older age of the nursing home residents. Another study also showed that older nursing home residents (mean age, 86 years) not dependent on HD showed weak immune responses to two doses of BNT162b2 [6]. Furthermore, we found that the median IgG titer against the anti-SARS-CoV-2 spike protein in the nursing home residents 2 weeks after the second dose was low (104 U/mL, IQR 11–492). We speculated that older age and undergoing HD affected their humoral response. According to previous studies from other countries, the median IgG titers among HD patients at 3 weeks after the second dose (median age, 67 years old) and healthy elderly people (median age, 83 years old) at 2–3 weeks were 171 U/mL [14] and 4030 U/mL [19], respectively.

Previous studies show that age has a negative impact on IgG levels after two doses of BNT162b2 among patients on HD [11, 12]; however, these reports did not cover elderly patients on HD who are more than 90 years old. Although age was correlated with anti-spike protein antibody levels in the control group, no such association was found for the nursing home residents in this study. This result could also be due to the wider age distribution in the controls. However, advanced age was associated with seronegativity even after the patient had received two BNT162b2 doses (Table 3). The seronegative nursing home residents showed lower serum albumin levels. Although serum albumin levels are known to be associated with immunity [20], this phenomenon might be associated with appetite loss due to older age (Table 3). Since the immune system deteriorates with age via cellular dysfunction due to decreased and malfunctioning lymphocytes, effective humoral responses to vaccines may not be expected in the elderly population [21].

HD patients who contract COVID-19 tend to show more rapid SARS-CoV-2 antibody decay than the general population, with the antibody titer decreasing at 4 weeks after seroconversion in some patients [3]. Conversely, another study showed that the seroconversion rate of patients on HD gradually increased after recovery from COVID-19 [22] or after receiving the BNT162b2 vaccine [23]. These results suggest that the immune responses of patients on HD may differ from those of the general population. Since it is well known that uremia can affect the immune system, as uremic toxins suppress the functions of white blood cells [24], natural and adaptive immunity may not work effectively to produce sufficient antibodies in a short period [25]. The impaired immune system in patients on HD cannot elicit optimal immune responses to vaccination, thus resulting in discrepancies in the immune response compared with that of the general population. Consequently, the HD population, especially elderly patients on HD, will have low titers of IgG, and the duration of appropriate IgG levels (if achieved) will be shorter than that of the general population. According to clinical trial data of mRNA-1273 vaccination in healthy volunteers, the IgG titer peaked at only 1 week after the second dose, irrespective of age [26], which was similar to the finding for the booster dose of BNT162b2 in a healthy population [27]. Those two studies showed that the IgG titers increased by more than ten times. In the present study, even though the anti-spike protein antibody levels in nursing home residents decreased to 83 U/mL (IQR 17–511) during the 2–3 weeks after the second dose, more than half of the nursing home residents showed an increase in their IgG levels during this period. In contrast, 84% of the control group showed a decrease in their IgG levels during the same time period. We could not find any difference between nursing home residents with slow humoral response and those without it in this study. However, these results further indicate that it may take a relatively long time for some elderly patients on HD to develop sufficient IgG levels following vaccination.

We only analyzed the levels of anti-spike protein antibodies as a humoral response; thus, cellular immune responses following COVID-19 vaccination should also be considered in patients on HD [9]. A previous study reported that HD patients with COVID-19 decreased the numbers of T cells, helper T cells, natural killer cells, and inflammatory cytokines compared with those of HD patients without COVID-19 or non-HD patients with COVID-19 [28]. Although a recent study showed that patients on HD could generate efficient T cell-dependent cellular immunity [29], there remains a concern that they might not develop appropriate cellular immunity against COVID-19 even after vaccination.

Although the anti-SARS-CoV-2 spike protein antibody has been widely used as a surrogate marker for monitoring immunization against COVID-19, the correlation between anti-spike antibody and neutralizing antibody levels has not been fully elucidated. One study showed that many dialysis patients who maintained a similar seropositivity rate to that of controls after two vaccine doses nevertheless contracted COVID-19 [30]. This might be associated with the lower titer of anti-spike protein antibodies and weak cellular immunity in dialysis patients. Thus, considering a third dose of BNT162b2 in these patients could be advantageous if the serum anti-spike protein antibody levels are below the level required to confer effective protection (29 U/mL) [31]. Although we considered an antibody level of 29 U/mL as the cut-off for neutralizing SARS-CoV-2 according to the literature, this value was based only on an in vitro analysis [14]. Therefore, the significance of anti-spike protein antibody levels in elderly patients on HD needs to be further investigated in a large prospective cohort study along with other metrics to assess effective immunization, such as neutralizing capability against viruses.

The average age of patients on HD has increased steadily in Japan [32], and as the population ages, it is expected that more elderly patients would require HD and enter nursing homes in the future. Although the patients included in this study may represent a relatively small portion of the population, they represent a crucial population to assure long-term protection against the spread of COVID-19, even after the pandemic ends.

This study has several limitations. Several differences between the nursing home residents and healthcare workers, such as age, hindered result interpretation. Ideally, nursing home residents not undergoing HD should have been included as a control, but the nursing home residents in Kokura-an were all on HD. The small number of nursing home residents did not provide sufficient statistical power to detect a significant correlation between the levels of anti-spike protein antibodies and other factors. Since all the patients on HD in this study reside in a particular nursing home, their backgrounds and other characteristics might have differed from those of other elderly patients on HD living in their own homes or other nursing homes. Moreover, since the optimal titer level (> 29 U/mL) considered in this study has not been confirmed in a clinical setting among patients on HD, further studies will be needed to determine the optimal IgG level required to prevent COVID-19 among elderly patients on HD.

In conclusion, elderly patients on HD did not show sufficient humoral responses to BNT162b compared to healthcare workers. Since patients receiving HD in HD centers and nursing home residents are relatively more vulnerable to COVID-19 than the general population due to their weak immunogenicity, more efforts for their protection are needed to implement suitable vaccination programs. COVID-19 outbreaks in medical and welfare facilities can be prevented only if medical facility users and healthcare workers are appropriately immunized.

## Supplementary Information

**Additional file 1.** **Table S1.** Difference in background characteristics of nursing home residents with and without effective IgG levels.

## Data Availability

The data underlying this article will be shared on reasonable request to the corresponding author.
