# Supplementary material for: Low humoral immune response to the BNT162b2 vaccine against COVID-19 in nursing home residents undergoing hemodialysis: a case–control observational study
Source: Ren Replace Ther. 2022 Mar 16;8(1):8. doi: 10.1186/s41100-022-00397-5 (PMC8924726; doi:10.1186/s41100-022-00397-5)
Supplement: Supplementary file 1 — Additional file 1. Table S1. Difference in background characteristics of nursing home residents with and without effective IgG levels. [file 41100_2022_397_MOESM1_ESM.docx]

**Supplementary Table 1. Difference in background characteristics of the nursing home residents with and without effective IgG levels.**

|  | **Effective IgG levels**  **(n = 18)** | **Deficient IgG levels**  **(n = 8)** | **p-value** |
| --- | --- | --- | --- |
| **Age (years)** | 86 (80–88) | 85 (73–98) | 0.60 |
| **Sex (male) (number, %)** | 6, 33% | 4, 50% | 0.42 |
| **BMI^1^ (kg/m^2^)** | 20.1 (17.7–22.2) | 19.0 (17.8–21.3) | 0.58 |
| **Dialysis vintage (months)** | 46 (25–122) | 85 (46–120) | 0.36 |
| **Diabetes mellitus (number, %)** | 4, 22% | 0, 0% | 0.07 |
| **Mean KT/V** | 1.55 (1.40–1.85) | 1.60 (1.35–1.85) | 0.98 |
| **White blood cell count (/μL)** | 5110 (4485–5765) | 5990 (5058–7278) | 0.07 |
| **Hemoglobin (g/dL)** | 10.7 (10.0–11.8) | 10.0 (9.5–10.9) | 0.15 |
| **Blood urea nitrogen (mg/dL)** | 52 (48–69) | 53 (42–63) | 0.45 |
| **Creatinine (mg/dL)** | 6.7 (6.3–7.7) | 6.6 (5.5–9.5) | 0.76 |
| **Albumin (g/dL)** | 3.2 (3.1–3.3) | 3.1 (2.8–3.5) | 0.48 |

^1^: body mass index
